# Design considerations when transitioning from paper case report forms (CRFS) to electronic data capture (EDC)

**DOI:** 10.1186/1745-6215-16-S2-P35

**Published:** 2015-11-16

**Authors:** Rebecca Lewis, Leona Batten, Charlotte Friend, Mark Webster-Smith, Stephanie Burnett, James Morden, Elizabeth Hill, Alexa Gillman, Sharon Ereira, Judith Bliss, Emma Hall, Claire Snowdon

**Affiliations:** 1 The Institute of Cancer Research Clinical Trials & Statistics Unit (ICR-CTSU), London, UK

## Background

ICR-CTSU introduced EDC in 2012; this necessitated a revision of the systems and processes implemented for paper CRFs. Traditionally sites manually completed, signed and posted CRFs to ICR-CTSU. Once received, CRFs were tracked and transcribed onto the study database by a member of the ICR-CTSU trial team. Databases contained multiple complex validations to aid central data management. Data queries were raised by ICR-CTSU and sent to the sites by post. These processes had to be revised when EDC was introduced and alteration of CRF and database design was integral to this.

## Implementation

Conventions for standard header and form submission fields have been developed. Form submission fields define when a CRF is considered ‘received’ by ICR-CTSU. Form design has been simplified and validations reduced in order to speed up remote data entry. Simple warnings are built into the study database to inform sites about data discrepancies in real time. Forms for trial visits are split into separate parts (eg lab values, scan results) in order that each section can be submitted separately when data are available. Continuous forms, with repeating question groups, are used where forms require updating at each visit (eg concomitant medication).

## Conclusion

When moving from CRFs to EDC, the differences between paper and electronic data capture must be considered when considering CRF design in order that data integrity can be maintained whilst providing a user-friendly interface for sites.

